# Efficacy and safety of cangrelor in patients with peripheral artery disease undergoing percutaneous coronary intervention – Insights from the CHAMPION program

**DOI:** 10.1016/j.ahjo.2021.100043

**Published:** 2021-08-25

**Authors:** J. Antonio Gutierrez, Robert A. Harrington, Gregg W. Stone, Ph. Gabriel Steg, C. Michael Gibson, Christian W. Hamm, Matthew J. Price, Renato D. Lopes, Sergio Leonardi, Jayne Prats, Efthymios N. Deliargyris, Kenneth W. Mahaffey, Harvey D. White, Deepak L. Bhatt, on Behalf of the CHAMPION Investigators

**Affiliations:** a Duke University Medical Center, Duke University School of Medicine, Durham, NC, USA; b Brigham and Women's Hospital Heart & Vascular Center, Harvard Medical School, Boston, MA, USA; c Stanford University Medical School, Stanford, CA, USA; d Columbia University Medical Center, The Cardiovascular Research Foundation, New York, NY, USA; e Université Paris-Diderot, Sorbonne Paris Cité, INSERM U-1148, DHU FIRE, Hópital Bichat, Assistance Publique-Hópitaux de Paris, Paris, France; f Beth Israel Deaconess Medical Center, Division of Cardiology, Boston, MA, USA; g Kerckhoff Heart and Thorax Center, Bad Nauheim, Germany; h Scripps Clinic and Scripps Translational Science Institute, La Jolla, CA, USA; i Duke Clinical Research Institute, Duke University School of Medicine, Durham, NC, USA; j Fondazione IRCCS Policlinico San Matteo, Pavia, Italy; k Elysis, Carlisle, MA, USA; l Science and Strategy Consulting Group, Basking Ridge, NJ, USA; m University of Auckland, Auckland City Hospital, Auckland, New Zealand

**Keywords:** Peripheral artery disease, Percutaneous coronary intervention, Antiplatelet therapy

## Abstract

**Background:**

Peripheral artery disease (PAD) is associated with an increased risk of ischemic events following percutaneous coronary intervention (PCI). More aggressive antiplatelet therapy may mitigate this risk. The present study evaluates the efficacy of cangrelor in patients with PAD undergoing PCI.

**Methods and results:**

This is a pooled analysis from the CHAMPION PCI, CHAMPION PLATFORM, AND CHAMPION PHOENIX trials, evaluating cangrelor versus either clopidogrel or placebo in PCI patients. The occurrence of the primary endpoint of death, myocardial infarction, or ischemia-driven revascularization (IDR) was assessed in patients with and without PAD. GUSTO severe bleeding at 48 h was also evaluated. There were 1720 (7%) patients with PAD and 22,802 (93%) without PAD. After adjustment for differences in baseline variables, PAD patients, compared with those without PAD, experienced increased odds of the primary endpoint (OR [95% CI] = 1.27 [0.91, 1.77], P = 0.16) and GUSTO severe bleeding (OR [95% CI] = 3.24 [1.28, 8.21], P = 0.01). In PAD patients, the primary endpoint was 4.7% with cangrelor vs. 7.2% with clopidogrel (OR [95% CI] = 0.64 [0.42,0.96]); in patients without PAD the primary endpoint was 3.5% with cangrelor vs. 4.2% with clopidogrel (OR [95% CI] = 0.83 [0.72,0.95]), P-interaction 0.23. Among patients with or without PAD, there was no significant difference in the rate of GUSTO severe bleeding with cangrelor compared with control, P-interaction 0.86.

**Conclusions:**

In a pooled analysis of the CHAMPION studies, PAD was associated with increased rates of ischemic and bleeding complications. Cangrelor reduced the odds of ischemic events, without increasing GUSTO severe bleeding.

**Clinical trial registration:**

clinicaltrials.gov identifiers: CHAMPION PCI (NCT00305162), CHAMPION PLATFORM

(NCT00385138), CHAMPION PHOENIX (NCT01156571)

## 1 Introduction

Identification of major determinants of ischemic risk among patients undergoing percutaneous coronary intervention (PCI) is essential for optimizing periprocedural therapy. Patients with peripheral artery disease (PAD) undergoing PCI, when compared with patients with coronary artery disease (CAD) alone, have been shown to experience increased rates of cardiovascular events. In light of such findings, it has been recommended that this complex patient population be targeted for more aggressive therapy [1], [2].

The novel intravenous P2Y_12_ receptor antagonist cangrelor has an immediate onset of action and a short half-life, 3 to 6 min. In a pooled analysis of the three CHAMPION (Cangrelor versus Standard therapy to Achieve Optimal Management of Platelet Inhibition) randomized, double blind double-dummy studies (CHAMPION PCI, CHAMPION PLATFORM, and CHAMPION PHOENIX), the intense antiplatelet effect provided by cangrelor at the time of PCI reduced the odds of ischemic events and stent thrombosis without a significant increase in severe bleeding or blood transfusions [3], [4], [5], [6]. In this patient-level analysis of the CHAMPION program, we explored the ischemic and bleeding risk associated with PAD and the efficacy and safety of cangrelor in patients with PAD undergoing PCI.

## 2 Materials and methods

The design and primary findings of the CHAMPION studies have been published previously [3], [4], [5], [7]. The present study combines individual patient data from the CHAMPION PCI, CHAMPION PLATFORM, and CHAMPION PHOENIX trials. The primary difference among the aforementioned phase 3 trials is the timing and loading dose of clopidogrel, patient population [PCI indication – stable angina, non-ST-elevation acute coronary syndrome (NSTE-ACS), or ST-elevation myocardial infarction (STEMI)], and primary efficacy outcome definitions (Fig. S1 Supplementary appendix).

### 2.1 Study patients

CHAMPION PCI and CHAMPION PHOENIX enrolled patients undergoing PCI for stable angina, NSTE-ACS, and STEMI. CHAMPION PLATFORM enrolled patients undergoing PCI for stable angina and NSTE-ACS. CHAMPION PLATFORM and CHAMPION PHOENIX prohibited the use of a P2Y_12_ inhibitor or abciximab 7 days prior to randomization and eptifibatide, tirofiban, or fibrinolytic therapy 12 h prior to randomization. The PAD subgroup was defined as patients for whom PAD was checked off in the electronic case report form. Inquiries in regard to the severity of lower extremity symptoms such as claudication or history of PAD related procedures (prior revascularization [endovascular or surgical] or amputation) were not made.

### 2.2 Study treatment

In all three CHAMPION trials, patients undergoing PCI were randomly assigned in a double blind, double-dummy fashion to receive cangrelor at a dose of 30 μg/kg bolus and 4 μg/kg per min infusion or matching placebo. Timing of study drug or matching placebo administration varied according to PCI indication. Patients undergoing PCI for unstable angina or NSTE-ACS received study drug or matching placebo as soon as possible following randomization and after coronary anatomy was determined. In STEMI patients, study drug or matching placebo could be administered without confirmation of coronary anatomy.

All patients randomized to cangrelor received 600 mg of clopidogrel at the end of study drug infusion. Among patients randomized to placebo, the timing and loading dose of clopidogrel varied among the three studies; in CHAMPION PCI, 600 mg of clopidogrel was given at the start of PCI; in CHAMPION PLATFORM, 600 mg was given at the end of PCI; and in CHAMPION PHOENIX, the timing (start or end of PCI) and loading dose of clopidogrel (300 mg or 600 mg) were determined by the site investigator.

### 2.3 Endpoints

The primary efficacy endpoint for the present study was a composite of death, myocardial infarction, and ischemia-driven revascularization (IDR). The secondary efficacy endpoint was a composite of the primary efficacy endpoint and stent thrombosis. Both composite endpoints and their individual components were evaluated at 48 h and 30 days after randomization. Myocardial infarction was defined by the second universal definition criteria [8]. Stent thrombosis was defined as angiographic stent thrombosis associated with IDR and/or by the Academic Research Consortium criteria [9], [10]. The primary safety endpoint was the Global Use of Strategies to Open Occluded Coronary Arteries (GUSTO) defined severe non coronary-artery bypass grafting (CABG) bleeding. Thrombolysis in Myocardial infarction (TIMI) and Acute Catheterization and Urgent Intervention Triage strategy (ACUITY) defined bleeding were also collected. All components of the efficacy composite endpoints were adjudicated. Bleeding events were derived from investigator reported data and were not adjudicated [11].

### 2.4 Statistical analysis

The primary efficacy analysis for the present study was conducted using a modified intention-to-treat population, comprised of patients with known PAD status, who received study drug, and underwent PCI. The safety analysis was conducted using patients who received study drug.

Heterogeneity among the CHAMPION PCI, CHAMPION PLATFORM, and CHAMPION PHOENIX trials was evaluated using the Breslow-Day test. Baseline characteristics were summarized according to PAD history and treatment, cangrelor vs. clopidogrel; and were analyzed using analysis of variance for continuous variables and the chi-square test for categorical variables. Treatment comparisons were performed as event proportions and presented as odds ratios (OR) with 95% confidence intervals (CI). Treatment with cangrelor was randomized with balanced distribution of baseline characteristics, therefore respective efficacy and safety analyses were not adjusted. The interaction between PAD and treatment effect was tested using the Breslow-Day method. The Kaplan-Meier method was used to construct time-to-event plots for the primary efficacy endpoint at 48 h following randomization and was compared using the log-rank test.

Efficacy and safety analyses were performed to assess the effect of PAD, compared with no PAD, with study treatment in the model. The aforementioned analyses were adjusted for the following variables: age, sex, diabetes mellitus, current smoking status, hypertension, hyperlipidemia, prior stroke/transient ischemic attack, prior myocardial infarction, prior PCI, prior CABG surgery, heart failure, indication (stable angina, NSTE ACS, or STEMI), antithrombotic regiment at time of PCI (clopidogrel loading dose, low-molecular-weight heparin, unfractionated heparin, fondaparinux, bivalirudin, or glycoprotein IIb/IIa inhibitor) stent type and number of vessels treated. Statistical analyses were performed using SAS software, version 9.3 (SAS institute, Cary, North Carolina).

## 3 Results

The CHAMPION trials randomized 25,384 patients. The modified intention-to-treat population with known PAD status comprised of 24,522 patients. There were 1720 (7%) patients with PAD at baseline and 22,802 (93%) without PAD. Baseline and procedural characteristics according to history of PAD are shown in Table 1. Patients with PAD were older in age, female, and more likely to have DM, hypertension, hyperlipidemia, prior history of stroke/TIA, myocardial infarction, revascularization (PCI or CABG), or heart failure, when compared with patients without PAD.

**Table 1:** Baseline characteristics: PAD vs. no PAD.

	PAD	No PAD	P-value
	N = 1720	N = 22,802	
Demographic			
Age-yr.			<0.0001
Median	68.0	63.0	
Interquartile range	61, 75	55, 71	
Female sex, *n* (%)	29.8	27.6	0.04
Weight - kg			
Median	83.0	83.0	0.93
Interquartile range	72, 95	72, 95	

Medical history, *n* (%)			
Diabetes mellitus	45.1	28.3	<0.0001
Current smoker	28.8	29.3	0.67
Hypertension	87.1	74.8	<0.0001
Hyperlipidemia	82.0	63.2	<0.0001
Prior stroke or TIA	12.2	4.6	<0.0001
Prior myocardial infarction	32.5	22.1	<0.0001
Prior PTCA or PCI	39.6	22.2	<0.0001
CABG	25.6	9.0	<0.0001
Heart failure	20.4	7.7	<0.0001

Procedural			
Indication^a^, *n* (%)			<0.0001
Stable angina	40.2	30.4	
NSTE ACS	52.7	57.7	
STEMI	7.2	12.0	

Antithrombotic, *n* (%)			
Aspirin^b^	93.7	93.7	0.97
Clopidogrel, loading dose (planned)			0.005
300 mg	9.3	11.5	
600 mg	90.7	88.5	
Low-molecular-weight heparin^b^	21.2	23.3	0.05
Unfractionated heparin^b^	71.2	74.3	0.005
Fondaparinux^b^	1.4	2.2	0.02
Bivalirudin^b^	31.2	24.7	<0.0001
Glycoprotein IIb/IIIa inhibitor	10.4	12.9	0.003

Stent type, *n* (%)			
Only drug eluting stent	920 (53.5)	11,451 (50.2)	0.009
Only bare metal stent	624 (36.3)	9473 (41.5)	<0.0001
Both	59 (3.4)	615 (2.7)	0.07
Neither	117 (6.8)	1263 (5.5)	0.03

PCI duration in mins	N = 1717	N = 22,793	0.885
Mean ± SD	24.9 ± 20.8	24.8 ± 20.3	
Median (Q1,Q3)	19.0 (10,33)	20.0 (11,32)	

Number of vessels treated			
Mean ± SD	1.2 ± 0.5	1.2 ± 0.4	0.003

PAD, peripheral artery disease; TIA, transient ischemic attack; PTCA, percutaneous transluminal coronary angioplasty; PCI, percutaneous coronary intervention; CABG, coronary artery bypass grafting; NSTE ACS, non ST-elevation acute coronary syndrome; STEMI, ST-elevation myocardial infarction.

a As determined by statistical analysis, taking into account clinical study data available after time of randomization.

b Prior or procedural.

Baseline and procedural characteristics for patients with and without PAD according to randomized treatment (cangrelor vs. clopidogrel) are displayed in Table 2. Both baseline and procedural characteristics were balanced among cangrelor and placebo cohorts in patients with PAD and without PAD.

**Table 2:** Baseline characteristics: cangrelor vs clopidogrel according to PAD history.

	PAD			No PAD		
	Cangrelor	Clopidogrel	P-value	Cangrelor	Clopidogrel	P-value
	N = 889	N = 831		N = 11,384	N = 11,418	
Demographic						
Age-yr.						
Median	68.0	68.0	0.96	63.0	63.0	0.40
Interquartile range	60, 75	61.75		55, 71	55, 71	
Female sex, *n* (%)	28.8	30.9	0.33	27.5	27.6	0.89
Weight – kg						
Median	83.0	83.6	0.48	83.0	83.0	0.97
Interquartile range	72, 94	72, 95		72, 95	72, 95	

Medical history, *n* (%)						
Diabetes mellitus	44.2	46.0	0.45	28.1	28.6	0.42
Current smoker	26.9	30.8	0.08	29.3	29.3	0.99
Hypertension	86.1	88.1	0.23	75.2	74.3	0.11
Hyperlipidemia	83.3	80.7	0.16	63.3	63.1	0.82
Prior stroke or TIA	10.9	13.6	0.09	4.7	4.4	0.18
Prior myocardial infarction	31.2	34.0	0.21	21.6	22.6	0.07
Prior PTCA or PCI	39.4	39.9	0.82	21.7	22.6	0.10
CABG	26.5	24.7	0.40	9.2	8.9	0.40
Heart failure	21.5	19.2	0.25	7.5	7.9	0.25

Procedural						
Indication, *n* (%)^a^			0.57			0.18
Stable angina	40.3	40.1		30.8	30.0	
NSTE ACS	52.0	53.4		57.6	57.7	
STEMI	7.8	6.5		11.6	12.3	

Antithrombotic, n (%)						
Aspirin^b^	93.5	93.9	0.74	94.0	93.3	0.02
Clopidogrel, loading dose (planned)			0.17			0.74
300 mg	10.2	8.3		11.4	11.6	
600 mg	89.8	91.7		88.6	88.4	
Low-molecular-weight heparin^b^	21.3	21.2	0.97	23.1	23.5	0.44
Unfractionated heparin^b^	71.7	70.8	0.68	74.2	74.4	0.74
Fondaparinux^b^	1.5	1.3	0.81	2.3	2.1	0.37
Bivalirudin^b^	29.5	33.0	0.12	24.6	24.8	0.73
Glycoprotein IIb/IIIa inhibitor	9.8	11.1	0.38	12.5	13.3	0.11

Stent type, *n* (%)			0.39			0.70
Only drug eluting stent	473 (53.2)	447 (53.8)		5729 (50.3)	5722 (50.1)	
Only bare metal stent	320 (36.0)	304 (36.6)		4697 (41.3)	4776 (41.8)	
Both	37 (4.2)	22 (2.6)		316 (2.8)	299 (2.6)	
Neither	59 (6.6)	58 (7.0)		642 (5.6)	621 (5.4)	

PCI duration in mins			0.14			0.57
Mean ± SD	24.2 ± 19.9	25.7 ± 21.8		24.8 ± 20.2	24.9 ± 20.4	
Median (Q1,Q3)	19.0 (10,32)	20.0 (11,33)		20.0 (11,32)	20.0 (11,32)	

Number of vessels treated			0.43			0.74
Mean ± SD	1.2 ± 0.5	1.2 ± 0.5		1.2 ± 0.4	1.2 ± 0.4	

PAD, peripheral artery disease; TIA, transient ischemic attack; PTCA, percutaneous transluminal coronary angioplasty; PCI, percutaneous coronary intervention; CABG, coronary artery bypass grafting; NSTE ACS, non ST-elevation acute coronary syndrome; STEMI, ST-elevation myocardial infarction.

a As determined by statistical analysis, taking into account clinical study data available after time of randomization.

b Prior or procedural.

## 4 Outcomes

### 4.1 PAD vs. no PAD

#### 4.1.1 48 hour post-randomization

**Fig. 1:**
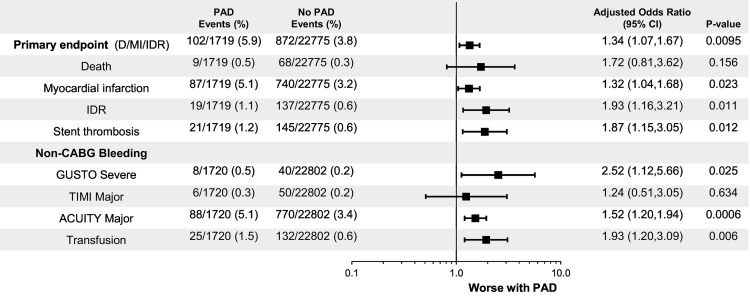
Primary efficacy and safety endpoints at 48 h in patients with and without PAD. The analyses were adjusted for the following variables: age, sex, diabetes mellitus, current smoking status, hypertension, hyperlipidemia, prior stroke/transient ischemic attack, prior MI, prior PCI, prior CABG surgery, heart failure, indication (SA, NSTE ACS, STEMI), loading dose, fondaparinux, glycoprotein IIb/IIa inhibitor, stent type and number of vessels treated. PAD, peripheral artery disease; MI, myocardial infarction; IDR, ischemia driven revascularization; ST, stent thrombosis; GUSTO, Global Use of Strategies to Open Occluded Arteries; TIMI, Thrombolysis in Myocardial Infarction; ACUITY, Acute Catheterization and Urgent Intervention Triage strategy; OR, odds ratio; CI, confidence interval.

Among patients with PAD the primary efficacy endpoint at 48 h was 5.9% vs. 3.8% without PAD (log rank P-value <0.001). Among patients with PAD the secondary efficacy endpoint at 48 h was 6.3% vs. 4.1% without PAD (log rank P-value <0.001). Figs. S2 and S3 (Supplementary appendix) depict the Kaplan-Meier estimates for the primary and secondary efficacy endpoints at 48 h for the PAD and no PAD cohorts. Patients with PAD experienced increased rates of the individual components of death, MI, IDR, or stent thrombosis. After adjustment for differences in baseline variables, patients with PAD had a statistically significant increased odds of the primary efficacy endpoint (adjusted OR [95% CI] = 1.34 [1.07, 1.67], P = 0.0095) and secondary efficacy endpoint (OR [95% CI] = 1.36 [1.10, 1.69], P = 0.005). Following adjustment, a history PAD at randomization was also associated with statistically non-significant increased odds of the individual components of death, MI, IDR, or stent thrombosis. (Fig. 1).

In the PAD cohort, the rate of GUSTO severe bleeding was 0.5% vs. 0.2% without PAD (Log Rank P-value = 0.009). Fig. S4 The rates of TIMI major, ACUITY major, and blood transfusion were also greater among patients with PAD vs. without PAD. (Fig. 1) After adjustment for differences in baseline variables, PAD continued to be associated with a significantly increased odds of GUSTO severe (adjusted OR [95% CI] = 2.52 [1.12, 5.66] P = 0.026), ACUITY major bleeding (adjusted OR [95% CI] = 1.52 [1.20, 1.94] P = 0.0006), and blood transfusion (adjusted OR [95% CI] = 1.93 [1.20, 3.09] P = 0.006).

#### 4.1.2 30 day post-randomization

**Fig. 2:**
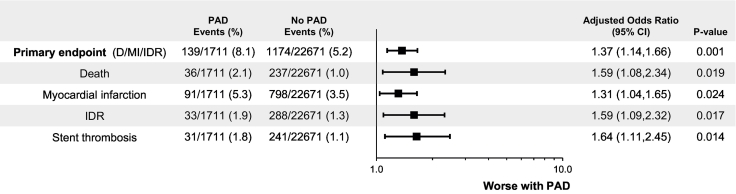
Primary efficacy endpoints at 30 days in patients with and without PAD. PAD, peripheral artery disease; MI, myocardial infarction; IDR, ischemia driven revascularization; ST, stent thrombosis; OR, odds ratio; CI, confidence interval. The analyses were adjusted for the following variables: age, sex, diabetes mellitus, current smoking status, hypertension, hyperlipidemia, prior stroke/transient ischemic attack, prior MI, prior PCI, prior CABG surgery, heart failure, indication (SA, NSTE ACS, STEMI), loading dose, fondaparinux, glycoprotein IIb/IIa inhibitor, stent type and number of vessels treated.

Among patients with PAD the primary efficacy endpoint at 30 days post-randomization was 8.1% vs. 5.2% without PAD (P = 0.0378). Among patients with PAD the secondary efficacy endpoint at 30 days post-randomization was 8.5% vs. 5.4% without PAD (P < 0.0001). The individual components of death, MI, IDR, or stent thrombosis at 30 days post-randomization were greater among patients with PAD vs. without PAD. After adjustment for differences in baseline variables, at 30 days patients with PAD was associated with a significant increased odds in the primary (adjusted OR [95% CI] = 1.38 [1.14, 1.68], P = 0.0009) and secondary efficacy endpoint (adjusted OR [95% CI] = 1.40 [1.16, 1.68], P = 0.0005). (Fig. 2).

### 4.2 Cangrelor vs. control

#### 4.2.1 48 hour post-randomization

**Fig. 3:**
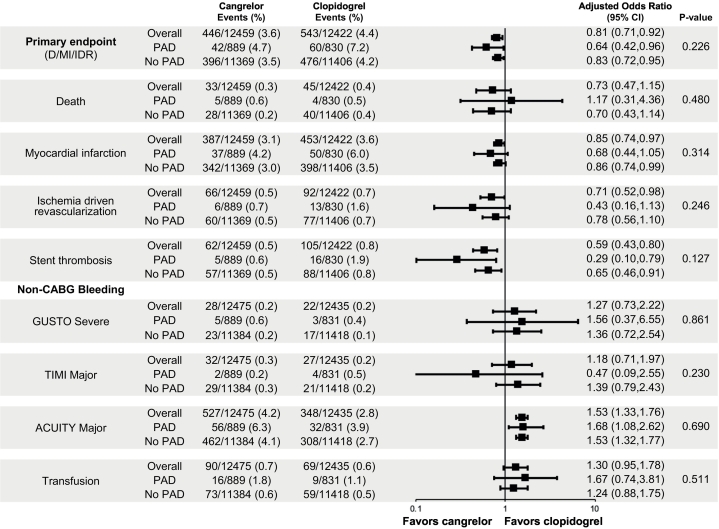
Primary efficacy and safety endpoints at 48 h of cangrelor versus clopidogrel according to PAD history. PAD, peripheral artery disease; MI, myocardial infarction; IDR, ischemia driven revascularization; ST, stent thrombosis; OR, odds ratio; CI, confidence interval.

**Fig. 4:**
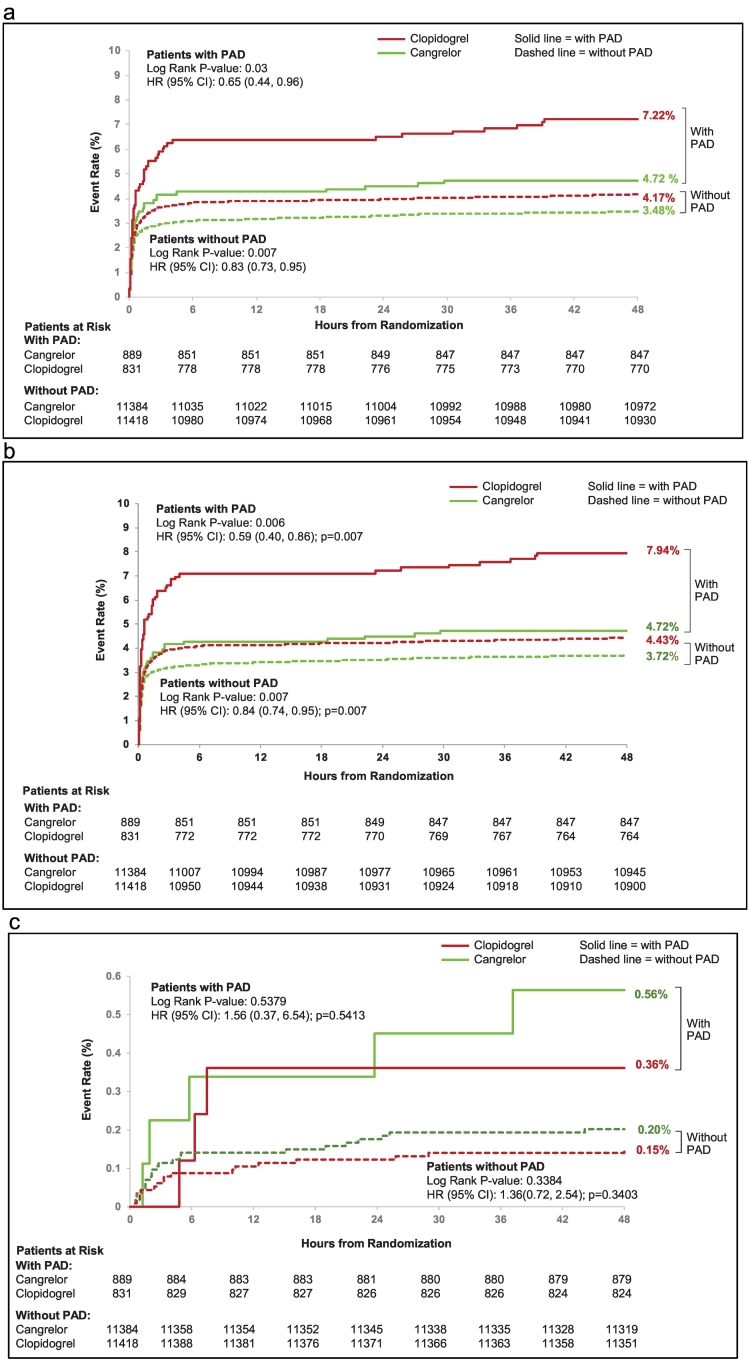
a. Kaplan Meier curves for primary efficacy endpoint at 48 h in patients with and without PAD (cangrelor vs. clopidogrel). PAD, peripheral artery disease. b. Kaplan Meier curves for secondary efficacy endpoint at 48 h in patients with and without PAD (cangrelor vs. clopidogrel). PAD, peripheral artery disease. c. Kaplan Meier curves for primary safety endpoint at 48 h in patients with and without PAD (cangrelor vs. clopidogrel). PAD, peripheral artery disease.

The effect of cangrelor on efficacy outcomes according to PAD history is depicted in Fig. 3. In the PAD cohort, the rate of the primary efficacy endpoint of death, myocardial infarction, and IDR at 48 h post-randomization was 4.7% with cangrelor vs. 7.2% with clopidogrel (OR [95% CI] = 0.64 [0.42, 0.96]); in patients without PAD the primary efficacy endpoint was 3.5% with cangrelor vs. 4.2% with clopidogrel (OR [95% CI] = 0.83 [0.72, 0.95]), P-interaction 0.23. In the PAD cohort, the rate of the secondary efficacy endpoint of death, myocardial infarction, IDR, and stent thrombosis at 48 hour post-randomization was 4.7% with cangrelor vs. 8.0% with clopidogrel (OR [95% CI] = 0.57 [0.39, 0.86]); in patients without PAD the secondary efficacy endpoint was 3.7% with cangrelor vs. 4.4% with clopidogrel (OR [95% CI] = 0.83 [0.73, 0.95]), P-interaction 0.08. Fig. 4a and b depict the Kaplan-Meier estimates for the primary and secondary efficacy endpoints at 48 h for patients with and without PAD according to treatment, cangrelor vs. clopidogrel. In regard to the individual components of the primary and secondary endpoints, at 48 h cangrelor exerted its greatest effect on stent thrombosis. Among patients with PAD, the individual component of stent thrombosis was 0.6% with cangrelor vs. 1.9% with clopidogrel (OR [95% CI] = 0.29 [0.10, 0.79]; in patients without PAD the rate of ST was 0.5% with cangrelor vs. 0.8% with clopidogrel (OR [95% CI] = 0.65 [0.46, 0.91]), P-interaction 0.13.

In both cohorts, with and without PAD, there was no significant difference in the rates of GUSTO severe (P-interaction 0.86), TIMI major bleeding (P-interaction 0.12), or blood transfusions (P-interaction 0.66) in patients treated with cangrelor compared with clopidogrel. (Fig. 4c) The rate of ACUITY major bleeding in the PAD cohort was 6.3% with cangrelor vs. 3.9% with clopidogrel (OR [95% CI] = 1.68 [1.08, 2.62]); and in patients without PAD was 4.1% with cangrelor vs. 2.7% with clopidogrel (OR [95% CI] = 1.53 [1.32, 1.77]), P-interaction 0.62.

#### 4.2.2 30 day post-randomization

**Fig. 5:**
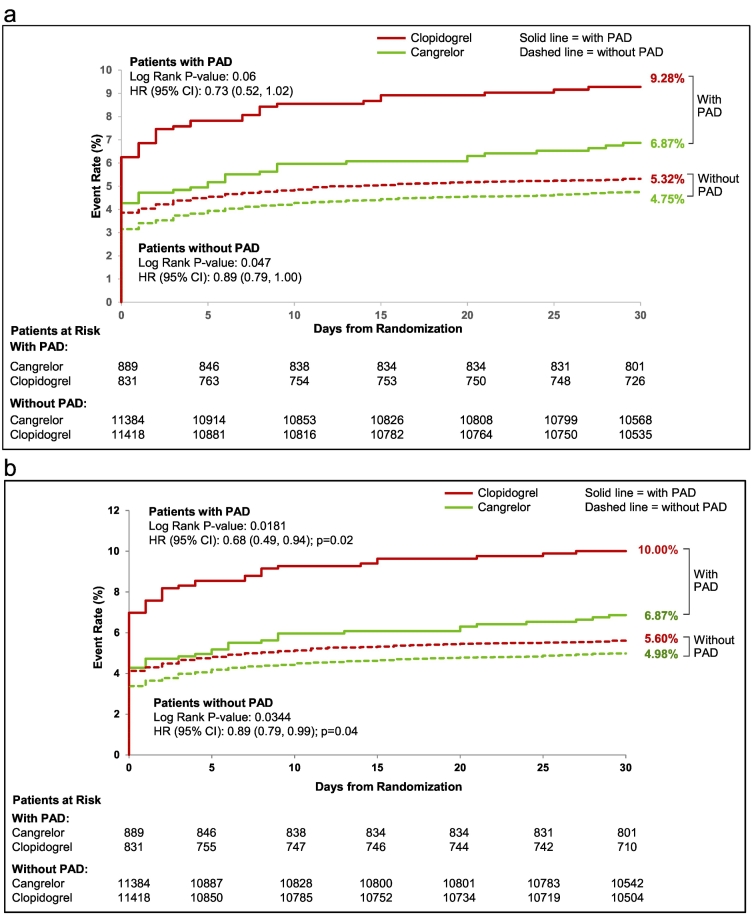
a. Kaplan Meier curves for primary efficacy endpoint at 30 days in patients with and without PAD (cangrelor vs. clopidogrel). PAD, peripheral artery disease. b. Kaplan Meier curves for secondary efficacy endpoint at 30 days in patients with and without PAD (cangrelor vs. clopidogrel). PAD, peripheral artery disease.

In the PAD cohort, the rate of the primary efficacy endpoint of death, myocardial infarction, and IDR, at 30-days post-randomization was 7.0% with cangrelor vs. 9.4% with clopidogrel (OR [95% CI] = 0.73 (0.51, 1.03)); in patients without PAD the primary efficacy endpoint was 4.9% with cangrelor vs. 5.5% with clopidogrel (OR [95% CI] = 0.89 [0.79, 1.00], P-interaction 0.28). (Fig. S5 Supplementary appendix) In the PAD cohort, the rate of the secondary efficacy endpoint of death, myocardial infarction, IDR, and stent thrombosis at 30-days post-randomization was 7.0% with cangrelor vs. 10.1% with clopidogrel (OR [95% CI] = 0.67 [0.47, 0.94]; in patients without PAD the secondary efficacy endpoint was 5.1% with cangrelor vs. 5.7% with clopidogrel (OR [95% CI] = 0.89 [0.79, 0.99], P-interaction 0.13). Fig. 5a and b depict the Kaplan-Meier estimates for the primary and secondary efficacy endpoints at 30 days for both PAD and non-PAD cohorts according to treatment, cangrelor vs. clopidogrel. In regard to the individual components of the primary and secondary endpoints at 30 days, cangrelor again exerted its greatest effect on stent thrombosis. Among patients with PAD, the individual component of stent thrombosis was 1.1% with cangrelor vs. 2.6% with clopidogrel (OR [95% CI] = 0.43 [0.20, 0.93]); in patients without PAD the rate of stent thrombosis was 0.9% with cangrelor vs. 1.2% with clopidogrel (OR [95% CI] = 0.75 [0.58, 0.96]), P-interaction 0.18.

## 5 Discussion

In the CHAMPION program, at 48 hour post-randomization the event rates of the primary (death, myocardial infarction, or IDR) and secondary composite endpoints (death, myocardial infarction, IDR, or stent thrombosis) among PAD patients undergoing PCI were 50% greater than patients without PAD. At 48 h, the observed increase in primary and secondary endpoint rates detected in the PAD cohort were driven primarily by increases in myocardial infarction, IDR, and stent thrombosis. With regards to safety, PAD patients, compared with those without PAD, experienced a >2-fold increase in rates of GUSTO severe bleeding and blood transfusion.

Patients with PAD undergoing PCI are at heightened risk for adverse events [12], [13]. For example, a study on revascularization outcomes pooling 1602 PAD patients from 8 randomized PCI trials found the presence of PAD to be associated with an increase risk in post procedure myocardial infarction [14]. The present study extends these findings to now include IDR and stent thrombosis. It has been postulated that the increased risk of cardiovascular events observed in PAD patients is due to the presence of multiple morbid conditions such as diabetes or polyvascular disease [12], [14], [15], [16], [17], [18]. In the current analysis, patients with PAD demonstrated higher rates of DM, stroke, prior coronary revascularization, and heart failure – supporting the aforementioned hypothesis.

An in depth evaluation of the secondary endpoints finds patients with PAD, in relation to those without, to experience a three-fold increase in stent thrombosis. This gives rise to the idea that the polyvascular disease phenotype of PAD and CAD may manifest a pathophysiology involving a severe thrombogenic environment in the PCI setting. This would explain the substantial efficacy of cangrelor in regard to stent thrombosis, as its maximal onset of action is immediate.

In the current pooled analysis of the three CHAMPION trials, cangrelor, consistent with the overall study results, reduced the odds of the primary composite endpoint of death, myocardial infarction, and IDR without a significant increase in GUSTO severe bleeding or blood transfusion among patients with and without PAD. Due to the heightened ischemic risk conferred by PAD, the consistent benefit of cangrelor in patients with and without PAD translated into a more robust absolute risk reduction in the primary (number needed to treat [NNT] of 47 at 48 h and 34 at 30 days) and secondary endpoints (NNT of 45 at 48 h and 33 at 30 days) among patients with a history of PAD. In both PAD and non-PAD cohorts, the use of cangrelor was not associated with a significant increase in GUSTO severe bleeding, TIMI major bleeding, or blood transfusion. However, when using the more sensitive ACUITY bleeding, cangrelor was associated with an increased risk of ACUITY major bleeding in patients with and without PAD.

There are limitations to the present study. First, the control groups differed in respect to the timing (start or end of procedure) and loading doses of clopidogrel (300 mg or 600 mg) among all 3 CHAMPION trials. The effect of cangrelor, however, is consistent throughout the 3 CHAMPION trials for all of the efficacy endpoints without significant heterogeneity [6]. Second, the studied populations varied regarding prior use of clopidogrel. CHAMPION PCI permitted prior clopidogrel use, whereas in CHAMPION PLATFORM and CHAMPION PHOENIX patients were clopidogrel naive. Lastly, the baseline characteristic of PAD was obtained through patient history without any specific testing such as ankle brachial index. This could result in a decrease in specificity of the diagnosis and the possibility that subjects with undiagnosed PAD were included in the no PAD group, though this would bias towards the null hypothesis. Furthermore, among PAD patients enrolled in the CHAMPION trials, the severity of PAD (such as claudication severity or critical limb ischemia) was not assessed. It has been shown that the treatment with potent antithrombotic therapies tends to provide the greatest benefit among patients with greatest disease burden [19]. As such, the lack of recorded PAD severity would only serve to diminish the treatment affect observed in the present study among PAD patients treated with cangrelor.

## 6 Conclusions

In a pooled analysis of the CHAMPION studies, cangrelor was associated with a lower risk of ischemic events, with no significant increase in severe bleeding or transfusion in patients with and without PAD. The reduction in ischemic outcomes seemed to be greater in patients with PAD treated with cangrelor. Our findings suggest that in PAD patients undergoing PCI, cangrelor might be a better option than clopidogrel.

## Sources of funding

The CHAMPION PHOENIX trial was funded by the sponsor at the time of the trial, 10.13039/100015237The Medicines Company. The Medicines Company (original sponsor) and Chiesi US, which currently markets cangrelor, funded the statistical analysis and the independent statistical validation.

## CRediT authorship contribution statement

**J. Antonio Gutierrez:** Writing – original draft, Writing – review & editing. **Robert A. Harrington:** Writing – review & editing. **Gregg W. Stone:** Writing – review & editing. **Ph. Gabriel Steg:** Writing – review & editing. **C. Michael Gibson:** Writing – review & editing. **Christian W. Hamm:** Writing – review & editing. **Matthew J. Price:** Writing – review & editing. **Renato D. Lopes:** Writing – review & editing. **Sergio Leonardi:** Writing – review & editing. **Jayne Prats:** Writing – review & editing. **Efthymios N. Deliargyris:** Writing – review & editing. **Kenneth W. Mahaffey:** Writing – review & editing. **Harvey D. White:** Writing – review & editing. **Deepak L. Bhatt:** Writing – review & editing, Supervision.

## Declaration of competing interest

The authors declare the following financial interests/personal relationships which may be considered as potential competing interests:

Dr. J. Antonio Gutierrez discloses the following – personal fees from Janssen Pharmaceuticals.

Dr. Robert A. Harrington discloses the following relationships – personal fees from Amgen Inc., Daiichi-Lilly, GILEAD Sciences, Gilead Sciences Inc., Janssen R and D, Medtronic, Merk, Novartis Corporation, The Medicines Company, Vida Health, Vox Media, and WebMD; Grants from AstraZeneca, BMS, CSL Behring, GSK, Merk, Portola, Sanofi-aventis, and The Medicines Company; ownership in Element Science and Myokardia; Advisory role in Evidint and Scandu.

Dr. Gregg W. Stone discloses personal fees from Boston Scientific, InspireMD, Atrium, Daiichi-Lilly, and Astra Zeneca.

Dr. Ph Gabriel Steg discloses the following relationships – Institutional research grant from Merck, Sanofi, and Servier; speaking or consulting fees from Amarin, AstraZeneca, Bayer, Boehringer-Ingelheim, Bristol-Myers-Squibb, CSL-Behring, Daiichi-Sankyo, GlaxoSmithKline, Janssen, Lilly, Merck Novartis, Pfizer, Regeneron, Sanofi, Servier, and The Medicines Company.

Dr. Charles Michael Gibson discloses personal fees from The Medicines Company.

Dr. Christian W. Hamm discloses the following relationships – Advisory board and speaking fees from AstraZeneca, Boehringer Ingelheim; speaking fees from Bayer, Daiichi Sankyo, Lilly, and Sanofi Aventis.

Dr. Matthew J. Price reports personal fees from The Medicines Company, during the conduct of the study, AstraZeneca, Merck & Co., Accriva Diagnostics, Boston Scientific, Medtronic, and Terumo.

Dr. Renato D. Lopes discloses the following relationships - Institutional research grant and consulting fees from Bristol-Myers Squibb; institutional research grant from GlaxoSmithKline; consulting fees from Bayer, Boehringer Ingleheim, Pfizer, Merck, Portola.

Dr. Sergio Leonardi discloses the following relationships - Advisory Board: The Medicine Company, AstraZeneca, and Eli Lilly.

Dr. Jayne Prats discloses the following relationships — was an employee of the Medicines Company at the time of the study and is a consultant for Chiesi USA.

Dr. Efthymios N. Deliagyris discloses the following relationships — was an employee of the Medicines Company.

Dr. Mahaffey discloses personal fees from American College of Cardiology, grants from Amgen, personal fees from AstraZeneca, personal fees from Bayer, personal fees from Boehringer Ingelheim, personal fees from Bristol Myers Squibb, personal fees from Cubist, grants from Daiichi, personal fees from Eli Lilly, personal fees from Epson, personal fees from Forest, personal fees from Glaxo Smith Kline, grants and personal fees from Johnson & Johnson, grants and personal fees from Medtronic, personal fees from Merck, personal fees from Mt. Sinai, personal fees from Myokardia, personal fees from Omthera, personal fees from Portola, personal fees from Purdue Pharma, personal fees from Spring Publishing, grants from St. Jude, grants from Tenax, personal fees from Vindico, personal fees from WebMD, outside the submitted work.

Dr. White discloses grants from Sanofi-Aventis, grants from Eli Lilly, grants from National Institute of Health, grants from GSK, grants from Merck Sharpe & Dohme, grants and personal fees from AstraZeneca, outside.

Dr. Deepak L. Bhatt discloses the following relationships - Advisory Board: Cardax, Elsevier Practice Update Cardiology, Medscape Cardiology, Regado Biosciences; Board of Directors: Boston VA Research Institute, Society of Cardiovascular Patient Care; Chair: American Heart Association Quality Oversight Committee; Data Monitoring Committees: Cleveland Clinic, Duke Clinical Research Institute, Harvard Clinical Research Institute, Mayo Clinic, Mount Sinai School of Medicine, Population Health Research Institute; Honoraria: American College of Cardiology (Senior Associate Editor, Clinical Trials and News, ACC.org), Belvoir Publications (Editor in Chief, Harvard Heart Letter), Duke Clinical Research Institute (clinical trial steering committees), Harvard Clinical Research Institute (clinical trial steering committee), HMP Communications (Editor in Chief, Journal of Invasive Cardiology), Journal of the American College of Cardiology (Guest Editor; Associate Editor), Population Health Research Institute (clinical trial steering committee), Slack Publications (Chief Medical Editor, Cardiology Today's Intervention), Society of Cardiovascular Patient Care (Secretary/Treasurer), WebMD (CME steering committees); Other: Clinical Cardiology (Deputy Editor), NCDR-ACTION Registry Steering Committee (Chair), VA CART Research and Publications Committee (Chair); Research Funding: Amarin, Amgen, AstraZeneca, Bristol-Myers Squibb, Chiesi (including for serving as co-Chair and co-PI of the CHAMPION trials), Eisai, Ethicon, Forest Laboratories, Ironwood, Ischemix, Lilly, Medtronic, Pfizer, Roche, Sanofi Aventis, The Medicines Company (including for serving as co-Chair and co-PI of the CHAMPION trials); Royalties: Elsevier (Editor, Cardiovascular Intervention: A Companion to Braunwald's Heart Disease); Site Co-Investigator: Biotronik, Boston Scientific, St. Jude Medical (now Abbott); Trustee: American College of Cardiology; Unfunded Research: FlowCo, Merck, PLx Pharma, Takeda.

## Appendix A Supplementary data

Supplementary material Image 1
