# Supplementary material for: Efficacy and safety of cangrelor in patients with peripheral artery disease undergoing percutaneous coronary intervention – Insights from the CHAMPION program
Source: Am Heart J Plus. 2021 Aug 25;9:100043. doi: 10.1016/j.ahjo.2021.100043 (PMC10978113; doi:10.1016/j.ahjo.2021.100043)
Supplement: Supplementary file 1 — Supplementary material [file mmc1.docx]

**Supplementary Appendix**

This appendix has been provided by the authors to give readers additional information about their work.

Supplement to:

Efficacy and Safety of Cangrelor in Patients with Peripheral Artery Disease Undergoing Percutaneous Coronary Intervention– Insights from the CHAMPION Program

J. Antonio Gutierrez, MD, MHS, Robert A. Harrington, MD, Gregg W. Stone, MD, Ph. Gabriel Steg, MD, C. Michael Gibson, MS, MD, Christian W. Hamm, MD, Matthew J. Price MD, Renato D. Lopes, MD, MHS, PhD, Sergio Leonardi, MD, Jayne Prats, PhD, Efthymios N. Deliargyris, MD, Kenneth W. Mahaffey, MD, Harvey D. White, DSc, Deepak L. Bhatt, MD, MPH, on Behalf of the CHAMPION Investigators*

*A full list of investigators can be found in Bhatt DL et al. NEJM 361(24):2330-41, 2009; Harrington RA et al. NEJM 361(24):2318-29, 2009; Bhatt DL et al. NEJM 368(14):1303-13, 2013.

**Efficacy and Safety of Cangrelor in Patients with Peripheral Artery Disease Undergoing Percutaneous Coronary Intervention– Insights from the CHAMPION Program**

**Contents**

**Figure S1: Summary of CHAMPION study designs and data analysis…………………...3**

**Figure S2: Kaplan Meier curves for primary efficacy endpoint at 48 hours in patients**

**with and without PAD………………………………...…….………….…....……....4**

**Figure S3: Kaplan Meier curves for secondary efficacy endpoint at 48 hours in patients**

**with and without PAD………………………………...…….………….…....……....5**

**Figure S4: Kaplan Meier curves for primary safety endpoint (GUSTO severe bleeding)**

**at 48 hours in patients with and without PAD.………………...…….……...…..6**

**Figure S5: Primary efficacy endpoint at 30 days in patients with and without PAD**

**(cangrelor vs. clopidogrel)…….……………………………..……….…....….…...7**

**Figure S1. CHAMPION studies and data analysis**

SA, stable angina; NSTE-ACS, non-ST-segment elevation acute coronary syndrome; STEM, ST-segment elevation myocardial infarction; PCI, percutaneous coronary intervention; IV, intravenous; MI, myocardial infarction; IDR, ischemia driven revascularization; ST, stent thrombosis; PAD, peripheral artery disease; GUSTO, Global Use of Strategies to Open Occluded Arteries; TIMI, Thrombolysis in Myocardial Infarction; ACUITY, Acute Catheterization and Urgent Intervention Triage strategy.

**Figure S2. Kaplan Meier curves for primary efficacy endpoint at 48 hours in patients with and without PAD.**

PAD, peripheral artery disease; HR, hazard ratio; CI, confidence interval.

**Figure S3. Kaplan Meier curves for secondary efficacy endpoint at 48 hours in patients with and without PAD.**

PAD, peripheral artery disease; HR, hazard ratio; CI, confidence interval.

**Figure S4 Kaplan Meier curves for primary safety endpoint (GUSTO severe bleeding) at 48 hours in patients with and without PAD.**

PAD, peripheral artery disease; HR, hazard ratio; CI, confidence interval.

**Figure S5. Primary outcomes at 30 days of cangrelor versus clopidogrel according to PAD history**

EP, endpoint; PAD, peripheral artery disease; MI, myocardial infarction; IDR, ischemia driven revascularization; ST, stent thrombosis; OR, odds ratio; CI, confidence interval.
